# 

**Published:** 1920-09-04

**Authors:** 


### Passing Events and Topics.

### News Trade Festival Dinner.

THE EARL OF ATHLONE has consented to preside at the Festival Dinner of the Newsvendors' Benevolent and Provident Institution, which will be held at the King's Hall, Holborn Restaurant, on Wednesday, October 20.

### War Memorial Tablets for Indian Villages.

To commemorate the services rendered during the Great War from various classes from which the Indian Army is recruited, and to form a memorial to those who lost their lives in the war, it has been decided to sanction the erection of memorial tablets in villages which furnished a considerable quota of recruits during the period 1914-1919.

### The Russian Red Cross in India.

LORD RONALDSHAY has received a letter from Her Royal Highness Princess Christian expressing thanks for Bengal's liberal response to the appeal made in India on behalf of the Russian Red Cross.

UNDER the will of the late Mr. J. W. Barclay £1,000 each has been left to the Manchester Royal Infirmary and the Salford Royal Hospital, and £500 each to the Ancoats Hospital and Ardwick and Ancoats Dispensary, the Manchester Hospital for Consumption, the Children's Hospital, Pendlebury, St. Mary's Hospital, Manchester, and the British Hospital, Buenos Aires. Mr. Barclay left £5,000 for a fund or funds for disabled soldiers.

### Matrons' and Sisters' Appointments.

ST. LUKE'S HOSPITAL, HALIFAX.—Miss Hope Ruby Willianott has been appointed night superintendent. She was trained at Bristol General Hospital and at Great Yarmouth Isolation Hospital, and has been sister at the South Devon Hospital and of the maternity ward at St. Luke's Hospital, Halifax.

THE ROYAL HOSPITAL, SHEFFIELD.—Miss Gertrude Spencer has been appointed *x*-ray sister. She was trained at the Institution for Nurses, Lincoln, and the Brownlow Hill Infirmary, Liverpool. Subsequently she did private nursing, and has been *x*-ray sister at the General Hospital, Birmingham, and at the Norfolk and Norwich Hospital.

DISTRICT HOSPITAL, NEAR ALFRETON, DERBYSHIRE.—Miss S. Maullin has been appointed matron. She was trained at West Bromwich and District Hospital, has been staff nurse at the Children's Hospital, Pendlebury, night sister and theatre sister at the Guest Hospital, Dudley, sister at the District Hospital, Dronfield, and sister at the Royal Naval Hospital, Haslar.

ISOLATION HOSPITAL, NORWICH.—Miss R. Menzies and Miss M. Dobson have been appointed sisters. Miss Menzies was trained at the Royal Alexandra Infirmary, Paisley, and has been matron of Turriff Red Cross Hospital. Miss Dobson was trained at Queen's Hospital, Birmingham, and at the City Hospital, Little Bromwich, becoming subsequently sister at the former hospital.

EAST RIDING MENTAL HOSPITAL, BEVERLEY.—Miss Agnes T. Brodie has been appointed matron. She was trained at the Kilmarnock Infirmary, and has been assistant matron of the District Asylum, Inverness.

BROMSGROVE OPEN-AIR SCHOOL FOR CRIPPLED CHILDREN, BIRMINGHAM.—Miss Marion Kennedy has been appointed sister-in-charge. She was trained at the Oldham Royal Infirmary, where she was later sister and night sister; she was subsequently sister at the Royal Liverpool Country Hospital for Children, Heswall, Cheshire.

WOLVERHAMPTON AND MIDLAND COUNTIES EYE INFIRMARY.—Miss M. J. Cannell has been appointed matron. She was trained at Sheffield Royal Infirmary, and has since been sister at Burton-on-Trent General Hospital, at the Stanley Hospital, Liverpool, at the Royal Westminster Ophthalmic Hospital, and matron of the Wolverhampton and Midland Counties Eye Infirmary. She has seen active service at home and abroad as a member of the Territorial Force Nursing Service, and has been a school nurse under Northampton Borough Education Authorities.

BURTON-UPON-TRENT ISOLATION HOSPITAL. — Miss Kate Boyes has been appointed matron. She was trained at Liverpool City Fever Hospital and Wolverhampton General Hospital, and has since been matron of Keynsham Isolation Hospital, of Farnfield Isolation Hospital and Sanatorium, sister at Ipswich Borough Isolation Hospital, at Boldon Fever Hospital, Sunderland, and at Bolton Borough Fever Hospital.

